# A multivariable model for perioperative transfusion risk in elective primary THA

**DOI:** 10.1186/s12891-026-09979-4

**Published:** 2026-06-09

**Authors:** Nikolai Ramadanov, Maximilian Voss, Maximilian Heinz, Dakota Fuchs, Robert Prill, Roland Becker

**Affiliations:** 1 Center of Orthopaedics and Traumatology, Brandenburg Medical School, University Hospital Brandenburg, Brandenburg/Havel, Germany; 2 Faculty of Health Science Brandenburg, Brandenburg Medical School Theodor Fontane, Brandenburg/Havel, Germany

**Keywords:** Total hip arthroplasty, Blood management, Transfusion, Risk stratification, Hemoglobin

## Abstract

**Background:**

Perioperative blood transfusion remains a relevant concern in elective total hip arthroplasty (THA), as it is associated with adverse outcomes and increased healthcare utilization. Reliable, procedure-specific risk stratification tools based on routinely available clinical data are needed to support individualized perioperative blood management.

**Methods:**

This retrospective cohort study included consecutive patients undergoing elective primary THA between 2016 and 2023 at a single certified arthroplasty center. Demographic data, comorbidity burden (ASA classification), perioperative laboratory values, operative characteristics, blood loss parameters, and transfusion data were extracted from a prospectively maintained registry. The primary outcome was perioperative allogeneic red blood cell transfusion. Univariable and multivariable logistic regression analyses were performed to identify independent predictors of transfusion. Model performance was assessed using receiver operating characteristic analysis and calibration testing.

**Results:**

A total of 648 patients were included, of whom 104 (16.0%) required perioperative transfusion. Transfused patients demonstrated lower preoperative hemoglobin levels, greater perioperative hemoglobin decline, and higher total and hidden blood loss. On multivariable analysis, lower preoperative hemoglobin, longer operative time, greater intraoperative blood loss, and lower body mass index were independently associated with transfusion, while male sex was inversely associated. Higher comorbidity burden (ASA ≥ III) showed a borderline association after adjustment. The final model demonstrated excellent discrimination with an area under the curve of 0.878 and good calibration.

**Conclusion:**

External validation of the present model is required before routine clinical application. Perioperative transfusion following elective primary THA can be predicted with good discriminative performance using routinely available clinical variables, supporting perioperative risk assessment and individualized blood management, although its applicability for preoperative patient counseling is limited.

**Level of evidence:**

Level III (retrospective cohort study).

**Supplementary Information:**

The online version contains supplementary material available at 10.1186/s12891-026-09979-4.

## Introduction

Total hip arthroplasty (THA) is among the most frequently performed and successful elective orthopedic procedures worldwide, with steadily rising numbers driven by an aging population and expanding indications [1]. Despite its generally excellent outcomes, THA carries substantial perioperative risks, including blood loss, transfusion requirements, and medical and surgical complications. Such events may compromise recovery, prolong hospitalization, and increase healthcare costs [2, 3].

Perioperative blood transfusion, in particular, has drawn increasing attention, as it is associated with adverse outcomes including postoperative infection, thromboembolic events, and elevated mortality risk [4, 5]. Although advances in surgical technique, anesthesia, and patient blood management have reduced transfusion rates, a significant proportion of patients undergoing THA still require allogeneic blood products. This underscores the importance of reliably identifying and refining transfusion risk throughout the perioperative course [6].

Accurate and robust risk stratification offers a potential way forward. By anticipating perioperative needs and transfusion risk, prediction models can support individualized perioperative planning, inform shared decision-making, and optimize healthcare resource utilization. In parallel, the increasing shift toward outpatient and resource-efficient arthroplasty care further underscores the need for precise perioperative optimization and risk stratification [7]. Several recent arthroplasty-focused studies have explored prediction of perioperative transfusion risk using combinations of demographic, hematologic, and procedural variables, but many available models remain limited by methodological weaknesses, lack of external validation, or insufficient procedure-specific focus [8].

While individual predictors such as preoperative hemoglobin or body mass index (BMI) have been described, comprehensive multivariable models that integrate diverse patient-specific and surgical factors are scarce. The development of a clinically practical, THA-specific multivariable model remains an unmet need.

The aim of this study was to develop a multivariable perioperative prediction model for elective THA to identify independent predictors of allogeneic blood transfusion and thereby support individualized perioperative planning and patient counseling. The model integrates both preoperative and intraoperative variables and is therefore intended for perioperative rather than purely preoperative risk estimation.

## Methods

### Study design and data source

This retrospective cohort study was conducted in accordance with the STROBE statement for observational studies. Data were derived from a prospectively maintained institutional arthroplasty registry at the University Hospital Brandenburg/Havel, a certified EndoCert arthroplasty center with standardized perioperative protocols.

All consecutive patients undergoing elective primary total hip arthroplasty (THA) between 1 January 2016 and 31 December 2023 were screened for eligibility. The present analysis represents a predefined study within the *Brandenburg THA Blood Management Series* [9–11], focusing on perioperative transfusion risk.

Ethical approval for registry-based retrospective analyses was granted by the local ethics committee (Ethics Committee of the Brandenburg Medical School Theodor Fontane, reference No. 292032025-BO-E-RETRO). The study was conducted in accordance with relevant guidelines and regulations and the principles of the Declaration of Helsinki (latest revision). The requirement for informed consent was waived due to the retrospective design and anonymized data processing.

### Patient selection

Inclusion criteria were: (1) elective primary THA, (2) age ≥ 18 years, (3) complete perioperative laboratory and clinical documentation. Exclusion criteria were: (1) revision THA, (2) fracture-related or oncologic indications, (3) simultaneous bilateral procedures, (4) incomplete key laboratory or transfusion data.

Although the registry is prospectively maintained, documentation of specific perioperative blood loss parameters was not mandatory throughout the entire study period and partly dependent on routine clinical documentation. As a result, a substantial proportion of cases had incomplete data for these variables and were excluded from the present analysis.

### Perioperative management and surgical technique

All procedures were performed under standardized institutional protocols regarding anesthesia, perioperative monitoring, thromboprophylaxis, and patient blood management. Surgical approach, implant selection, and cement usage were documented prospectively in the registry. Operative time and intraoperative blood loss were recorded as part of routine perioperative documentation. Intraoperative blood loss was documented based on the anesthesia record, including suction volume after subtraction of irrigation fluids and standardized estimation from surgical swabs. This approach followed institutional documentation protocols. Perioperative management protocols, including anemia workup, tranexamic acid administration, and transfusion practices, were standardized at the institutional level and remained largely consistent throughout the study period.

### Surgical technique

A modified anterolateral (Watson-Jones) approach [12] was consistently applied. Fixation type (cemented vs. uncemented) was selected based on patient-specific factors, including age and bone quality. Cementation, when performed, followed a standardized third-generation technique. Perioperative management, including anticoagulation handling, was conducted according to institutional protocols.

### Data collection and variables

The following variables were extracted from the registry and electronic medical records: (1) Demographics: age (years), sex; (2) Anthropometrics: body mass index (BMI, kg/m²); (3) Comorbidity burden: American Society of Anesthesiologists (ASA) physical status classification; (4) Preoperative laboratory values: hemoglobin (g/L), hematocrit (L/L); (5) Intraoperative variables: operative time (minutes), cement usage (yes/no), estimated intraoperative blood loss (mL); (6) Postoperative laboratory values: hemoglobin and hematocrit at 48 h postoperatively; (7) Transfusion data: occurrence of allogeneic red blood cell transfusion (yes/no) and total number of packed red cell (PRC) units administered; (8) Blood loss calculations: total blood loss and hidden blood loss, calculated using Nadler’s formula for total blood volume and the hemoglobin balance method. These calculations incorporate perioperative hemoglobin changes and transfusion volumes and may therefore be influenced by transfusion itself. Hemoglobin and hematocrit were assessed at 48 h postoperatively, as this time point more reliably reflects cumulative perioperative blood loss after hemodynamic equilibration [5].

### Outcomes

The primary outcome was the requirement for perioperative allogeneic red blood cell transfusion, defined as any allogeneic red blood cell transfusion administered during the index hospitalization. Transfusion decisions followed a restrictive institutional strategy and were generally considered at hemoglobin levels < 70 g/L or < 80 g/L in the presence of clinical symptoms of anemia, particularly in patients with relevant comorbidities such as cardiovascular disease or reduced physiological reserve (e.g., elderly patients). Symptoms were not strictly predefined but reflected routine clinical assessment. Transfusion practice remained consistent throughout the study period. Secondary outcomes included: (1) total number of transfused PRC units and (2) total blood loss and hidden blood loss.

### Statistical analysis

Continuous variables were assessed for normality using visual inspection of histograms and the Shapiro–Wilk test. Normally distributed data are presented as mean ± standard deviation, and non-normally distributed data as median with interquartile range. Categorical variables are reported as absolute counts and percentages.

Univariable analyses were performed to identify potential predictors of transfusion. Variables with clinical relevance or a univariable association at *p* < 0.10 were entered into multivariable logistic regression models to identify independent predictors. In total, 8 candidate predictors were initially assessed in univariable analyses. Of these, 6 variables were entered into the final multivariable model. Preoperative hematocrit and cemented fixation were not retained in the initial variable selection process due to collinearity with hemoglobin and limited additional predictive contribution; however, cemented fixation was reintroduced in the final model for clinical interpretability. Given 104 transfusion events, this corresponded to approximately 17 events per variable.

Multicollinearity was assessed using variance inflation factors (VIF), with values > 5 considered indicative of relevant multicollinearity. Continuous variables were entered into the regression models as continuous predictors without categorization. Linearity in the logit was assessed by visual inspection of partial residual plots and was considered acceptable for all continuous predictors. Model discrimination was evaluated using the area under the receiver operating characteristic curve (AUC), and calibration was assessed using the Hosmer–Lemeshow goodness-of-fit test, calibration intercept and slope, and visual inspection of the calibration plot.

All statistical analyses were performed using IBM SPSS Statistics version 28 (IBM Corp., Armonk, NY, USA). A two-sided p value < 0.05 was considered statistically significant.

## Results

### Study population

Of 1545 screened patients undergoing elective primary total hip arthroplasty, 648 were included in the final analysis. A total of 897 cases were excluded due to incomplete perioperative blood loss data. To assess potential selection bias, baseline characteristics of the included and excluded patients were compared. No statistically significant differences were observed regarding age, sex, BMI, ASA classification, preoperative hemoglobin, preoperative hematocrit, or fixation type (Supplementary Table 1). The included patient cohort covered a broad perioperative risk spectrum, with the majority of patients classified as ASA II or III. Baseline demographic, clinical, and perioperative characteristics are summarized in Table 1.

**Table 1:** Demographic, perioperative, and laboratory characteristics of the study cohort, stratified by transfusion status

Variable	Overall	No transfusion	Transfusion
Age, years	70.8 ± 10.3	70.0 ± 10.2	75.1 ± 10.0
BMI, kg/m²	29.7 ± 10.4	30.1 ± 11.1	27.9 ± 5.3
Preoperative hemoglobin, g/L	138.0 ± 13.7	140.2 ± 12.2	126.8 ± 15.8
Preoperative hematocrit, L/L	0.4 ± 0.0	0.4 ± 0.0	0.4 ± 0.0
Operative time, min	72.0 ± 23.0	69.4 ± 18.5	85.5 ± 35.9
Intraoperative blood loss, mL	530.3 ± 311.1	486.7 ± 264.9	758.0 ± 418.7
Hemoglobin at 48 h, g/L	104.6 ± 14.9	107.1 ± 13.9	91.4 ± 12.6
Hematocrit at 48 h, L/L	0.3 ± 0.0	0.3 ± 0.0	0.3 ± 0.0
Hemoglobin drop (preop to 48 h), g/L	33.4 ± 12.3	33.1 ± 11.2	35.4 ± 17.0
Total blood loss, mL	1372.3 ± 504.8	1343.0 ± 462.1	1524.8 ± 668.3
Hidden blood loss, mL	854.2 ± 443.0	858.0 ± 412.6	834.3 ± 578.4
Male sex, N(%)	275 (42.5%)	244 (44.9%)	31 (29.8%)
Cemented fixation, N(%)	100 (15.5%)	67 (12.3%)	33 (31.7%)
ASA II, N(%)	335 (51.8%)	302 (55.6%)	33 (31.7%)
ASA ≥ III, N(%)	273 (42.2%)	205 (37.8%)	68 (65.4%)

Patient demographics, comorbidity burden, perioperative variables, and laboratory parameters for the overall cohort, stratified by transfusion status where applicable

*Abbreviations*: *BMI* Body mass index, *ASA* American Society of Anesthesiologists

### Transfusion rate and blood loss

**Fig. 1:**
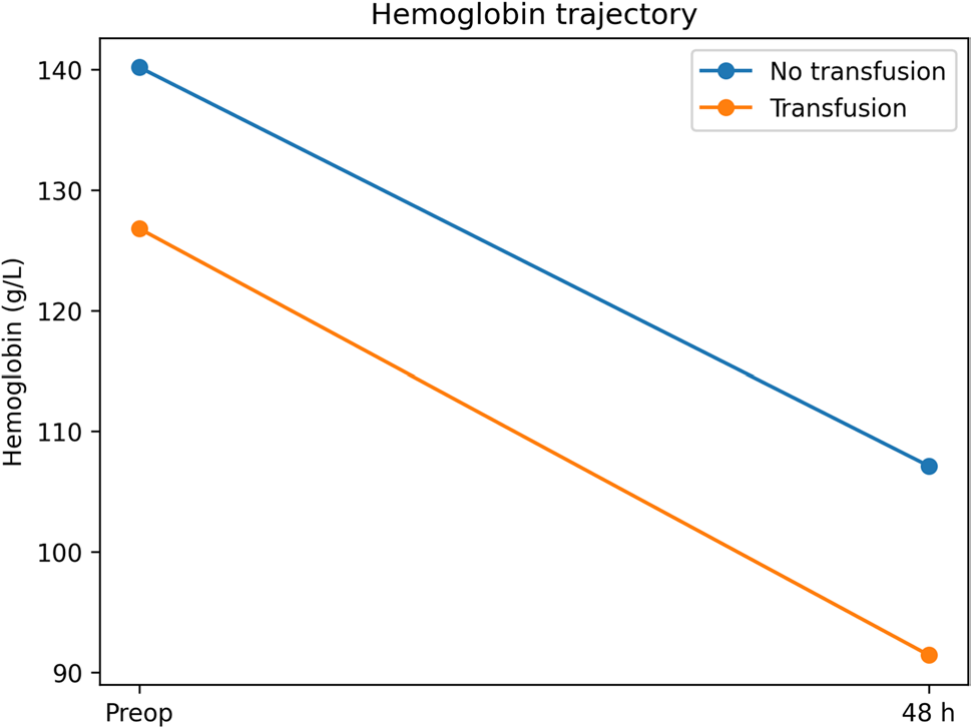
Perioperative hemoglobin trajectory. Mean hemoglobin levels preoperatively and at 48 h postoperatively, stratified by transfusion status

Overall, 104 patients (16.0%) required perioperative allogeneic red blood cell transfusion. Among transfused patients, the median number of packed red cell units administered was 2 (interquartile range [IQR] 2–3), ranging from 1 to 13 units. Preoperative hemoglobin and hematocrit values were lower in transfused patients, and a more pronounced postoperative decline at 48 h was observed. The perioperative hemoglobin trajectory from preoperative baseline to 48 h postoperatively is illustrated in Fig. 1. Calculated blood loss parameters were higher in transfused patients; however, these findings should be interpreted cautiously, as total blood loss and hidden blood loss are partly derived from postoperative hemoglobin changes and transfusion volumes.

### Univariable predictors of transfusion

On univariable analysis, transfusion was associated with higher age, higher ASA class, lower preoperative hemoglobin, longer operative time, greater intraoperative blood loss, and cemented fixation. Sex and body mass index also showed associations with transfusion risk on univariable testing. Detailed univariable analyses are presented in Table 2.

**Table 2:** Univariable predictors of perioperative blood transfusion

Predictor	OR	CI_low	CI_high	*p*
Age (per year)	1.056	1.032	1.081	< 0.001
Male sex (vs. female)	0.520	0.331	0.819	0.005
BMI	0.935	0.895	0.977	0.002
ASA 2 vs. I	1.311	0.383	4.492	0.666
ASA 3 vs. I	3.980	1.188	13.340	0.025
Preoperative hemoglobin (per g/L)	0.927	0.910	0.944	< 0.001
Operative time (per min)	1.026	1.017	1.035	< 0.001
Cemented fixation (vs. uncemented)	3.302	2.032	5.367	< 0.001
Intraoperative blood loss (per mL)	1.002	1.002	1.003	< 0.001

Univariable logistic regression analysis identifying factors associated with perioperative allogeneic blood transfusion

*Abbreviations* *BMI* Body mass index, *ASA* American Society of Anesthesiologists, *OR* Odds ratio, *CI* Confidence interval

### Multivariable risk stratification model

**Fig. 2:**
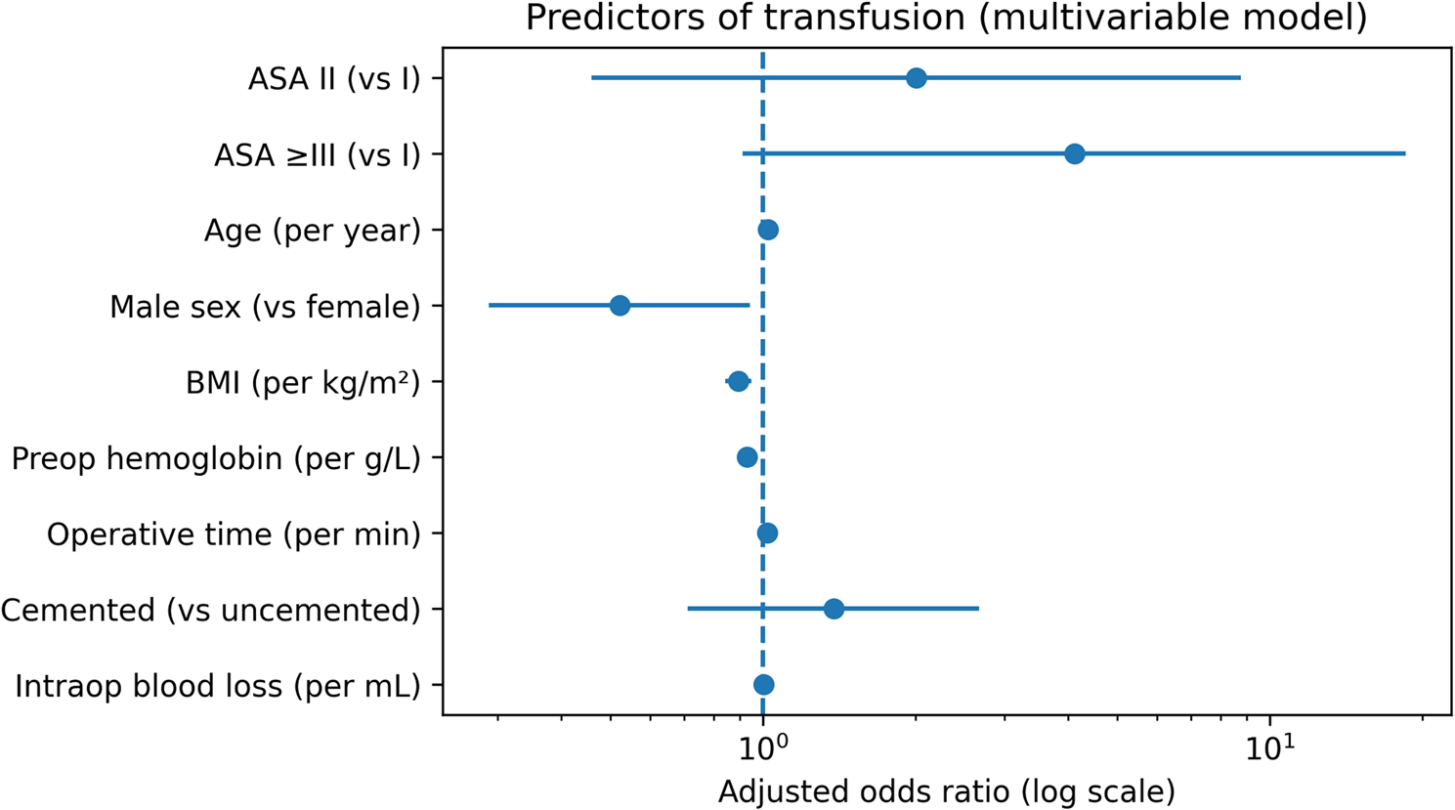
Multivariable predictors of transfusion. Forest plot showing adjusted odds ratios and 95% confidence intervals from the multivariable logistic regression model

**Fig. 3:**
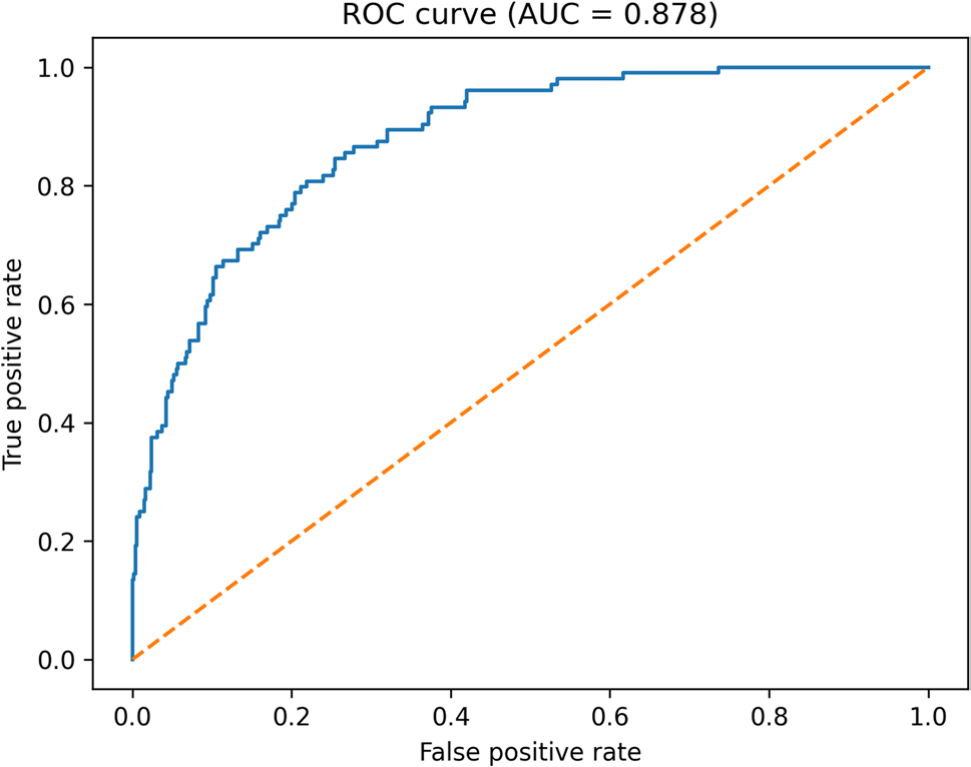
Discriminative performance of the transfusion risk model. Receiver operating characteristic (ROC) curve of the final multivariable model with corresponding area under the curve (AUC)

In the multivariable logistic regression model, independent predictors of perioperative transfusion included lower preoperative hemoglobin, longer operative time, greater intraoperative blood loss, and body mass index. Male sex was inversely associated with transfusion risk. After adjustment, higher comorbidity burden (ASA ≥ III) demonstrated a borderline association with transfusion requirement. Adjusted odds ratios with 95% confidence intervals for all included predictors are displayed in Fig. 2 and Table 3. The final model showed excellent discriminative performance, with an apparent (uncorrected) area under the receiver operating characteristic curve (AUC) of 0.878 (Fig. 3, Table 3). Model calibration was assessed using the Hosmer–Lemeshow goodness-of-fit test and visual inspection of the calibration plot.

**Table 3:** Multivariable logistic regression model for transfusion risk

Predictor	β	Adjusted OR	CI_low	CI_high	*p*
Intercept	6.762				
ASA II (vs. I)	0.696	2.006	0.459	8.759	0.355
ASA ≥ III (vs. I)	1.414	4.115	0.913	18.539	0.066
Age (per year)	0.024	1.024	0.993	1.056	0.125
Male sex (vs. female)	-0.673	0.521	0.288	0.944	0.032
BMI (per kg/m²)	-0.125	0.895	0.844	0.948	< 0.001
Preop hemoglobin (per g/L)	-0.075	0.930	0.910	0.951	< 0.001
Operative time (per min)	0.022	1.021	1.008	1.035	0.002
Cemented (vs. uncemented)	0.321	1.378	0.711	2.672	0.342
Intraoperative blood loss (per mL)	0.003	1.003	1.002	1.004	< 0.001

Regression coefficients (β), adjusted odds ratios (OR), and 95% confidence intervals are presented. Predicted probabilities can be calculated using the logistic regression equation based on the reported coefficients

*Abbreviations*: *BMI* Body mass index, *ASA* American Society of Anesthesiologists, *OR* Odds ratio, *CI* Confidence interval

## Discussion

The present study demonstrates that perioperative transfusion following elective primary total hip arthroplasty can be predicted with good discriminative performance using a limited set of routinely available preoperative and intraoperative variables. Within this single-center cohort, the multivariable model showed excellent discrimination and good calibration, supporting its potential utility for perioperative risk stratification and individualized blood management within the perioperative setting. Importantly, the model is not designed as a purely preoperative tool but rather as a perioperative prediction model that incorporates intraoperative information to refine risk assessment.

Preoperative hemoglobin emerged as the strongest independent predictor of transfusion. This finding is consistent with the central role of baseline hematologic reserve in determining transfusion need and underscores the importance of structured preoperative anemia screening and optimization. Even modest reductions in preoperative hemoglobin translated into a substantially increased transfusion risk, highlighting a potentially modifiable target within perioperative care pathways.

Operative time and intraoperative blood loss were also independently associated with transfusion. These parameters likely reflect both surgical complexity and cumulative exposure to bleeding risk. While intraoperative blood loss is not fully predictable preoperatively, its strong association with transfusion emphasizes the relevance of meticulous surgical technique, optimized exposure, and adherence to blood-sparing strategies throughout the procedure.

Body mass index demonstrated an independent association with transfusion risk, with lower BMI linked to higher transfusion probability. This may reflect lower circulating blood volume and reduced physiologic reserve. However, the association between body mass index and transfusion risk in total hip arthroplasty is not consistent across the literature, with some studies reporting higher transfusion rates in obese patients. Therefore, this finding should be interpreted with caution.

Male sex was inversely associated with transfusion requirement after multivariable adjustment. While this may partly reflect higher baseline hemoglobin concentrations and greater circulating blood volume, the persistence of this association despite adjustment for preoperative hemoglobin suggests that additional, unmeasured factors related to physiological reserve or blood volume may contribute, rather than sex itself acting as a direct causal factor.

After adjustment, higher comorbidity burden (ASA ≥ III) demonstrated a borderline association with transfusion requirement (adjusted OR 4.12, 95% CI 0.91–18.54, *p* = 0.066). While higher ASA status clearly correlated with transfusion on univariable analysis, its attenuated effect in the multivariable model suggests that much of this risk is mediated through measurable factors such as anemia, operative duration, and blood loss. This finding supports the integration of objective laboratory and procedural variables rather than reliance on global risk scores alone. The strong discriminative performance of the model indicates that reliable perioperative transfusion risk stratification is feasible using routine data. However, discriminative performance alone does not establish clinical utility. Formal assessment of clinical usefulness, such as decision curve analysis or evaluation of clinically relevant risk thresholds, was beyond the scope of the present study and should be addressed in future validation studies. The present findings are consistent with our previously published analyses within the Brandenburg THA Blood Management Series, which collectively indicate that while surgical and implant-related factors influence measured blood loss, transfusion risk is primarily determined by patient-specific hematologic reserve and physiological response to perioperative stress [9–11].

The present multivariable model integrates these determinants into a unified predictive framework for perioperative transfusion risk. It should be emphasized that the present model was developed for prediction rather than causal inference. Accordingly, variables such as operative time and intraoperative blood loss should not be interpreted as causal determinants of transfusion, but rather as predictive markers within the evolving perioperative course. Their associated odds ratios reflect predictive contribution within the model and should not be overinterpreted mechanistically.

Several limitations warrant consideration. (1) The retrospective single-center design may limit generalizability, and transfusion thresholds were determined by institutional practice rather than a fixed protocol. (2) Additionally, rare high-risk subgroups, such as ASA IV patients, were underrepresented, necessitating cautious interpretation in these populations. (3) Blood loss estimates based on hemoglobin balance methods may be influenced by perioperative transfusion and should therefore be interpreted with caution, particularly when comparing transfused and non-transfused patients. (4) The complete-case approach and exclusion of patients with incomplete blood loss data may introduce selection bias if missingness was not random. However, missing data were primarily related to documentation of perioperative blood loss rather than predefined patient characteristics. (5) Although perioperative protocols were largely standardized, minor temporal variations in clinical practice over the study period cannot be fully excluded. (6) Furthermore, no internal validation using bootstrapping or optimism correction was performed. Although model calibration was additionally explored using calibration intercept, slope, and visual assessment, the reported performance may still be subject to optimism and requires validation in independent cohorts. (7) In addition, variable selection based on univariable p values may introduce bias and increase the risk of overfitting, potentially inflating model performance estimates.

## Conclusion

External validation of the present model is required before routine clinical application. Perioperative transfusion following elective primary THA can be predicted with good discriminative performance using a limited set of routinely available clinical variables. This multivariable model may support perioperative risk assessment and individualized blood management, although its applicability for preoperative patient counseling is limited.

## Supplementary Information

Supplementary Material 1. Supplementary Table S1: Comparison of baseline characteristics between included and excluded patients. Baseline demographic and clinical characteristics of patients included in the final analysis and those excluded due to incomplete perioperative data are presented. Continuous variables are reported as mean ± standard deviation and compared using independent samples t-tests. Categorical variables are presented as counts (percentages) and compared using chi-square tests. Preoperative hemoglobin and hematocrit were not sufficiently available in the excluded cohort, as incomplete laboratory data constituted part of the exclusion criteria; therefore, no statistical comparison was performed for these variables. Abbreviations: BMI, body mass index; ASA, American Society of Anesthesiologists; NR, not reported.

## Data Availability

The datasets used and/or analyzed during the current study are available from the corresponding author on reasonable request.
