# Supplementary material for: A multivariable model for perioperative transfusion risk in elective primary THA
Source: BMC Musculoskelet Disord. 2026 Jun 9;27:502. doi: 10.1186/s12891-026-09979-4 (PMC13248325; doi:10.1186/s12891-026-09979-4)
Supplement: Supplementary file 1 — Supplementary Material 1. Supplementary Table S1: Comparison of baseline characteristics between included and excluded patients. Baseline demographic and clinical characteristics of patients included in the final analysis and those excluded due to incomplete perioperative data are presented. Continuous variables are reported as mean ± standard deviation and compared using independent samples t-tests. Categorical variables are presented as counts (percentages) and compared using chi-square tests. Preoperative hemoglobin and hematocrit were not sufficiently available in the excluded cohort, as incomplete laboratory data constituted part of the exclusion criteria; therefore, no statistical comparison was performed for these variables. Abbreviations: BMI, body mass index; ASA, American Society of Anesthesiologists; NR, not reported. [file 12891_2026_9979_MOESM1_ESM.docx]

| Variable | Included (n=648) | Excluded (n=897) | p-value |
| --- | --- | --- | --- |
| Age, years | 70.8 ± 10.3 | 69.9 ± 10.6 | 0.12 |
| Male sex | 275 (42.5%) | 398 (44.4%) | 0.05 |
| BMI, kg/m² | 29.7 ± 10.4 | 29.4 ± 10.1 | 0.60 |
| ASA ≥III | 273 (42.2%) | 386 (43.0%) | 0.78 |
| Preoperative hemoglobin, g/L | 138.0 ± 13.7 | NR | - |
| Preoperative hematocrit, L/L | 0.4 ± 0.0 | NR | - |
| Cemented fixation | 100 (15.5%) | 133 (14.8%) | 0.71 |
